# Core–Shell Silk Fibroin Hydrogel Microneedles Functionalized with Antibody-Binding Domains for Transdermal Delivery

**DOI:** 10.3390/biomimetics10120798

**Published:** 2025-11-27

**Authors:** Min Ki Lee, Ae Sol Lee, Chang Sup Kim

**Affiliations:** 1 Graduate School of Biochemistry, Yeungnam University, Gyeongsan 38541, Republic of Korea; dlalsrl1220@gmail.com; 2 Department of Chemical & Biochemical Engineering, Dongguk University, Seoul 04620, Republic of Korea; saol12@dgu.ac.kr

**Keywords:** antibody binding domain, silk fibroin, core–shell structure, hydrogel microneedle patch, transdermal delivery

## Abstract

Microneedle (MN) patches comprise a promising platform for transdermal delivery of macromolecular therapeutics. However, achieving sufficient mechanical strength for skin penetration while maintaining high biocompatibility and efficient antibody loading remains a major challenge. In this study, we designed and developed a core–shell-structured hydrogel MN patch composed of a silk fibroin core and a protein-based shell layer for antibody loading and potential transdermal release. The latter was constructed using a fusion protein consisting of the B and C domains of *Staphylococcus aureus* protein A (BC) and a tyrosine-rich mussel adhesive protein (MAP), thereby enabling antibody binding via the BC domains. By harnessing biomimetic design strategies, the BC-MAP shell facilitates antibody immobilization via specific affinity interactions, while the silk fibroin core provides substantial mechanical strength: the MN patch demonstrated a penetration force approximately 4.2 times greater than that required to pierce porcine skin. Collectively, our core–shell-structured hydrogel MN patch is a promising platform for transdermal antibody delivery.

## 1. Introduction

Microneedle (MN) patches have been widely investigated as an efficient local transdermal delivery system due to their minimal invasiveness and excellent drug penetration ability, while avoiding systemic metabolism [1,2,3]. Conventional MNs have been fabricated from inorganic materials such as silicon, titanium, and stainless steel, as well as synthetic polymers [4]. However, these materials suffer from several limitations, including insufficient biocompatibility, potential inflammation and infectious issues during long-term use, and inadequate biodegradability [5]. To overcome these drawbacks, recent studies have focused on hydrogel-based MN patches composed of naturally derived biomaterials.

Hydrogel-based MN patches can readily encapsulate therapeutic agents and control their release through the patches’ porous matrix [6]. Moreover, the patches swell upon insertion to ensure firm attachment to the skin and can be entirely removed after use [5,7]. However, their limited mechanical strength often hinders efficient skin penetration, thereby constraining their potential for clinical applications [8].

Natural biopolymer silk fibroin (SF) is widely used as a primary material for hydrogel-based MN fabrication due to its biocompatibility and high mechanical properties [9,10]. SF-based MNs have been successfully fabricated to achieve skin penetration while maintaining structural integrity [11]. However, high concentrations of SF cause densely packed networks with reduced pore size, which significantly hinders the release of large macromolecules such as antibodies [12]. This trade-off between mechanical properties and drug release is a key challenge in the development of SF-based hydrogel MN systems.

The B and C domains of *Staphylococcus aureus* protein A, which can be utilized as antibody-binding regions that reversibly interact with immunoglobulins, have been utilized in antibody purification processes and in biosensor platforms to facilitate the immobilization of antibodies [13,14]. However, their use in drug delivery systems is rarely reported. Due to their distinct binding affinities toward antibodies of various isotypes and species, the interaction with these domains and pre-immobilized antibodies, such as immune checkpoint inhibitors, can be weakened by engagement with endogenous antibodies. Hence, the controlled release of surface-bound antibodies can be introduced.

**Figure 1:**
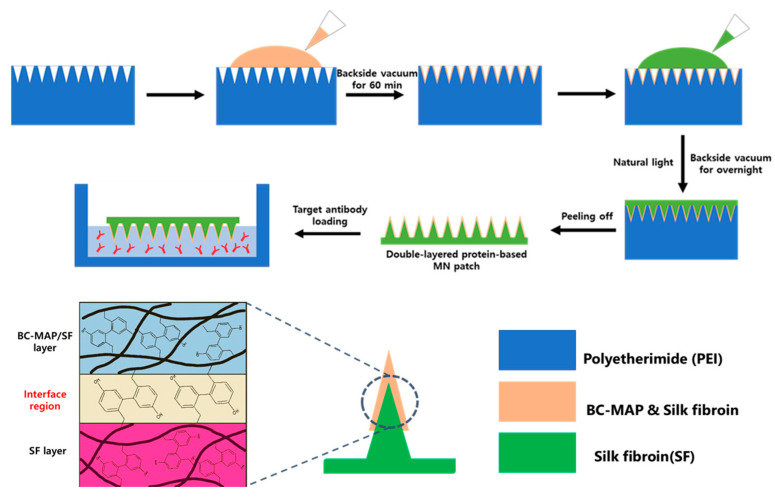
Schematic illustration of BC-MAP@SF core–shell hydrogel microneedle patch fabrication.

Biomimetic designs and structures have increasingly guided the development of advanced microneedle systems, where natural materials and structures inspire improved mechanical performance and biological functionality [15]. In the present work, we developed a core–shell hydrogel MN patch that combines the mechanical durability of SF with the reversible antibody-binding capability of the B and C domains of protein A (Figure 1). The SF-based core, composed of a higher concentration of silk fibroin, provides a dense network that ensures sufficient rigidity for effective skin penetration. In contrast, the shell incorporates a lower SF concentration blended with the functionalized BC-MAP protein, forming a more hydrated and porous matrix that allows efficient antibody binding and potential competitive release in response to endogenous antibodies. By spatially separating these two roles, the core–shell design prevents the trade-off typically observed in single-component hydrogels, allowing the microneedles to maintain high mechanical strength while still supporting effective loading and controlled release of antibodies. To achieve this integration, the B and C domains were genetically fused to a tyrosine-rich mussel adhesive protein (MAP) to provide BC-MAP, which was subsequently crosslinked with SF through bioinspired di-tyrosine coupling. The resulting MN patch demonstrated excellent mechanical strength and biocompatibility, thereby making it a promising platform for the efficient transdermal delivery of antibodies.

## 2. Materials and Methods

### 2.1. Materials

Codon-optimized BC-MAP (Y13W and Y79W) and BC-MAP (Y13F and Y79F) genes were synthesized by Genescript (Piscataway, NJ, USA). Expression vector pET22b(+) and *Esherichia coli* strain BL21 (DE3) were purchased from Novagen (Darmstadt, Germany). Luria–Bertani (LB) broth, sodium phosphate monobasic (NaH_2_PO_4_), sodium chloride (NaCl), and all other reagents were purchased from Sigma-Aldrich (Saint Louis, MO, USA). Unless otherwise specified, the purity of the chemicals used is 98% or higher. Polyclonal anti-rabbit immunoglobulin (Ig)G (alkaline phosphatase) was purchased from Sigma-Aldrich. Mouse IgG1, IgG2a, IgG2b, and IgG3 were purchased from Biolegend (Sandiego, CA, USA), and anti-mouse CTLA-4 was purchased from BioXcell (Lebanon, PA, USA). An SR7000 gold sensor slide planar Ni-NTA chip was purchased from Reichert (Depew, NY, USA). Dulbecco’s Modified Eagle’s Medium (DMEM) was purchased from Gibco (Saint Louis, MO, USA). Fetal bovine serum (FBS) and 1% streptomycin were purchased from Hyclone (Marlborough, MA, USA). Cell counting kit-8 (CCK-8) was purchased from Dojindo (Kumamoto, Japan).

### 2.2. Construction of Expression Vectors

To construct the expression vector, we designed MAP-fused BC domains with mutations at Y13 and Y79. From the N-terminus, the B and C domains were used sequentially, and two GGGS linkers were inserted between each domain. Subsequently, the sequence of MAP (fp-5), along with two GGGG linkers, was inserted after the C domain. For mutation, two tyrosine residues (Y13 and Y79) in the BC domains were substituted with tryptophan and phenylalanine, respectively, resulting in two distinct genes. The synthesized genes were digested with BamH1 and Nco1 restriction enzymes and subsequently ligated into the *Bam*HI and *Nco*I sites of the pET22b(+) vector to construct pET-BC-MAP (Y13W and Y79W) and pET-BC-MAP (Y13F and Y79F). The sequences of the BC domains and MAP were used from previously reported studies.

### 2.3. Expression and Purification of BC-MAP Fusion Protein

The recombinant plasmids containing pET-BC-MAP (Y13W and Y79W) and pET-BC-MAP (Y13F and Y79F) were transformed into *E. coli* BL21 (DE3). The transformants were cultured until reaching an optical density of 0.8–0.9 at 600 nm in LB medium containing 50 μg/mL ampicillin at 37 °C. Afterward, 1 mM of isopropyl β-D-1-thiogalactopyranoside was added to induce protein expression. After 10 h of incubation at 37 °C, the cells were harvested via centrifugation at 4000× *g* for 10 min, after which the cell pellets were resuspended in lysis buffer (50 mM NaH_2_PO_4_, 300 mM NaCl, 10 mM imidazole, 1 mM dithiothreitol, and 1 mM phenylmethylsulfonyl fluoride; pH 8.0). The resuspended cells were sonicated using a sonic dismembrator (Q500; Qsonica, Farmingdale, NY, USA), after which the soluble and insoluble fractions were separated via centrifugation at 10,000× *g* for 10 min. Subsequently, the soluble fraction was purified using Ni-NTA affinity chromatography, and then the purified proteins were eluted with elution buffer (50 mM NaH_2_PO_4_, 300 mM NaCl, 250 mM imidazole, pH 8.0). The purity and concentration of the purified proteins were analyzed by using sodium dodecyl sulfate (SDS)-polyacrylamide gel electrophoresis (PAGE) and the Bradford assay, respectively. Finally, the purified proteins were dialyzed against deionized water and then lyophilized.

### 2.4. Antibody Binding Affinity of BC-MAP Proteins

Dot blot analysis was performed to evaluate the antibody binding ability of BC-MAP (Y13W and Y97) and BC-MAP (Y13F and Y79F). To this end, each protein was applied to a nitrocellulose membrane and dried for 1 h at room temperature. The membrane was then blocked for 1 h with TBS solution (50 mM Tris and 150 mM NaCl; pH 7.4) containing 5% (*w*/*v*) skimmed milk. After blocking, the membrane was washed three times with TTBS solution (TBS with 0.05% (*v*/*v*) Tween-20; pH 7.4), followed by treatment with alkaline phosphatase-conjugated polyclonal anti-rabbit IgG. After 1 h at room temperature, the membrane was washed twice with TTBS and once with TBS. Finally, the membrane was developed using nitro blue tetrazolium/5-bromo-4-chloro-3-indolylphosphate substrate for dot blot visualization.

We used a surface plasmon resonance (SPR) SR7500DC system (Reichert, Depew, NY, USA) for the qualitative analysis of binding affinity. Initially, 30 μg/mL of BC-MAP (Y13W and Y97) and BC-MAP (Y13F and Y79F) were applied to the SR7000 gold sensor chip with a planar Ni-NTA surface to immobilize the ligand, after which 1X PBS (pH 7.4) containing 1 mg/mL BSA was used to block the sensor chip. Different concentrations of mouse IgGs (IgG1, IgG2a, IgG2b, and IgG3) and anti-mouse CTLA-4 were injected into the SPR sensor for 3 min, followed by rinsing for 7 min with 1X PBS (pH 7.4). Regeneration was performed with 350 mM EDTA buffer (pH 8.0) to completely dissociate the antibody. Data were processed and analyzed using the Scrubber 2 program. Association rate constant (*ka*) and dissociation rate constant (*kd*) values were calculated by fitting data from individual experiments, after which equilibrium dissociation constant (*K_D_*) values were determined using the following equation:

*K_D_* = *kd*/*ka*. (1)

### 2.5. Fabrication and Characterization of the BC-MAP@SF Core–Shell Hydrogel MN Patch

To this end, 0.01% (*w*/*v*) of BC-MAP (Y13W and Y79W) and BC-MAP (Y13F and Y79F) were mixed with 20% (*w*/*v*) SF in 50 mM sodium acetate (pH 5.5). Subsequently, to form the shell layer of the hydrogel MN patch, 1 mM tris(bipyridine)ruthenium(II) chloride ([Ru(II)bpy_3_]^2+^) and 30 mM sodium persulfate (SPS) were added to the mixed BC-MAP and SF solutions. The mixture was then poured into a polydimethylsiloxane (PDMS) mold and kept under a back–side vacuum system at −85 kPa for 10 min. Next, to create the core layer, 50% (*w*/*v*) SF with 1 mM [Ru(II)bpy_3_]^2+^ and 30 mM SPS was added to the PDMS mold and kept under a back–side vacuum at −85 kPa for an additional 6 h. The fabrication process was performed under fluorescent light to induce di-tyrosine crosslinking between the tyrosine residues in the proteins (BC-MAP and SF), as previously described in [11]. To visualize the two distinct layers in the MN patch, rhodamine B and FITC-dextran were then added to the shell and core layers, respectively.

The core–shell structure of the fabricated MN patch was confirmed using bright-field and fluorescence microscopy (Leica, Wetzlar, Germany). To evaluate the antibody binding property of the fabricated MN patch, it was incubated overnight at room temperature with an Alexa350-antibody solution (Thermo Fisher Scientific, Waltham, MA, USA). After incubation, the MN patch was thoroughly washed at least three times with 1X PBS buffer to remove unbound antibodies. Finally, the Alexa350-antibody-MN patch was observed using a fluorescence microscope.

The swelling property of the hydrogel MN patch was tested in vitro. To this end, it was incubated in 1X PBS (pH 7.4) for 0, 3, 4, and 10 min at room temperature. Morphological changes in the MNs were observed using a bright-field microscope (Leica). To distinguish the pore size between the core and shell layers, the MN patch was lyophilized for 2 h and examined via high-resolution field-emission scanning electron microscopy (SEM; JSM-7401F; JEOL, Akishima, Japan). To measure the fracture force of the MNs, compression tests were performed using a universal testing machine (Instron, Norwood, MA, USA) equipped with a 10 kN load cell. MN patches (10 × 10 MNs) were mounted on a stainless-steel base plate, after which the moving sensor was applied perpendicular to the axis of the MN patch at a constant speed of 0.025 mm/s. Force was recorded upon contact with the top point of the MN patch, and the fracture point was identified as the maximum force just before a sudden drop in the compressive force. The fracture force for each MN was calculated by dividing the maximum force by the number of MNs. The experiments were repeated four times on different samples.

### 2.6. In Vitro Cytocompatibility Assay

Following the biological evaluation of medical devices in vitro cytotoxicity testing (ISO 10993-5) [16], BC-MAP- and SF-based hydrogels were incubated with DMEM containing 10% FBS and 1% streptomycin for 24 h at 37 °C to obtain hydrogel extracts with a ratio of 0.1 g/mL. At the same time, 1 × 10^3^ HaCaT (human keratinocyte) and NIH3T3 (mouse fibroblast) cells were seeded in each well. After 24 h, the supernatants were replaced with hydrogel extract and incubated for 24 and 72 h at 37 °C under 5% CO_2_. Cell viability was measured using the CCK-8 assay, with blank medium and 15% DMSO being used as the negative and positive controls, respectively. Furthermore, the LIVE/DEAD kit (Calcein-AM for cytoplasmic staining of living cells/EthD-1 for nuclear staining of dead cells, Thermo Fisher Scientific, Waltham, MA, USA) was employed to visually distinguish viable and dead cells, and the results were analyzed by fluorescence microscope (BX60; Olympus, Tokyo, Japan).

## 3. Results and Discussion

### 3.1. Production and Antibody Binding Affinity of BC-MAP Proteins

**Figure 2:**
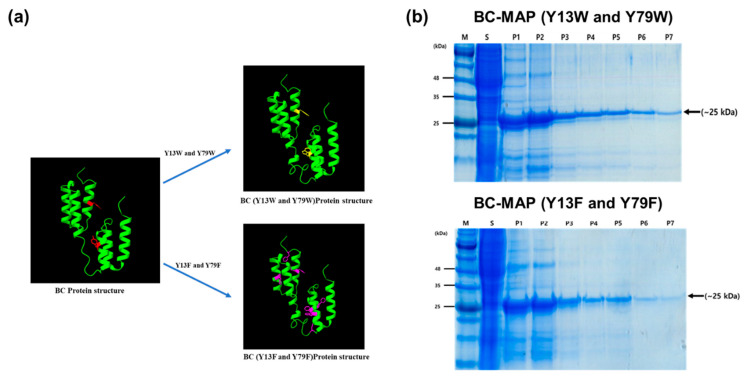
Production of MAP-fused BC domain. (**a**) Structural images of the BC domain and its mutants. The colored amino acids indicate the mutation sites. (**b**) SDS-PAGE analysis results of purified BC-MAP mutants using Coomassie blue staining.

To produce the protein-based hydrogel with antibody binding affinity, we genetically fused the B and C domains from *S. aureus* protein A with MAP. The di-tyrosine reaction is a bioinspired crosslinking system that chemically conjugates tyrosine residues and forms a 3D network [17,18]. MAP is a tyrosine-rich protein that has been used as both a hydrogel and a natural adhesive through di-tyrosine crosslinking [19]. To avoid structural changes in the B and C domains during the di-tyrosine crosslinking, we replaced the tyrosine residue in each domain with another aromatic amino acid: tryptophan and phenylalanine for BC-MAP (Y13W and Y79W) and BC-MAP (Y13F and Y79F), respectively. Subsequently, we simulated the structural differences in the BC-MAP proteins using ChimeraX and noted that no significant differences were observed between the BC-MAP protein and the two mutants [20] (Figure 2a).

**Figure 3:**
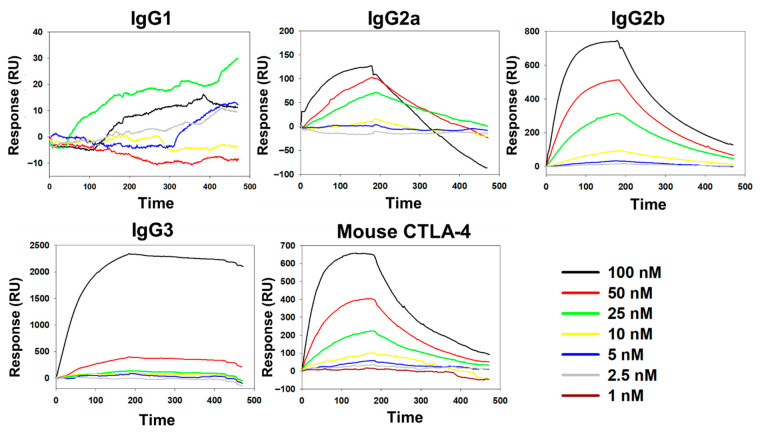
SPR analysis results of BC-MAP (Y13W and Y79W) towards different isotypes of antibodies (Mouse IgG1, IgG2a, IgG2b, IgG3 and CTLA-antibody).

**Figure 4:**
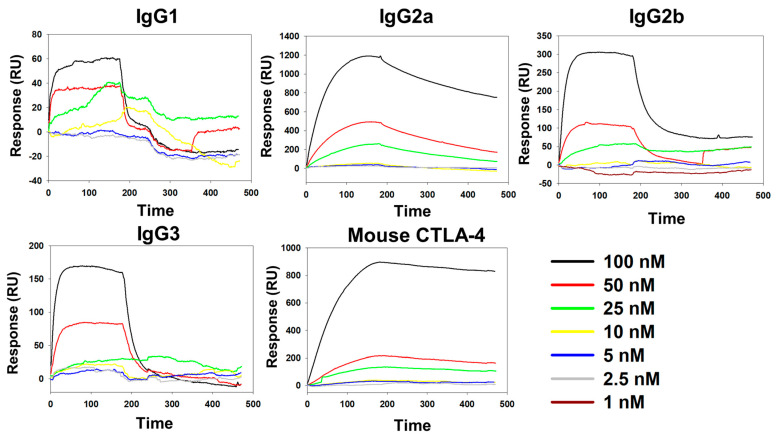
SPR analysis results of BC-MAP (Y13F and Y79F) towards different isotypes of antibodies (Mouse IgG1, IgG2a, IgG2b, IgG3 and CTLA-antibody).

**Figure 5:**
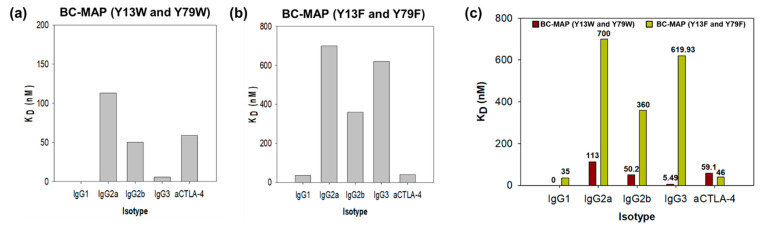
Calculated KD value of (**a**) BC-MAP (Y13W and Y79W) and (**b**) BC-MAP (Y13F and Y79F) mutants to different isotypes. (**c**) Comparison of the KD value of two BC-MAP mutants to different isotypes. BC-MAP (Y13W and Y79W) exhibited more minor differences in affinity between isotypes.

BC-MAP (Y13W and Y79W) and BC-MAP (Y13F and Y79F) were successfully produced and purified (Figure 2b). Protein A can interact with immunoglobulin while exhibiting different binding affinities to its isotypes [21]. To determine whether the BC domain retained its characteristic when the tyrosine residues were replaced with structurally similar amino acids (tryptophan and phenylalanine), we analyzed the antibody binding affinity of the two mutant BC-MAP proteins; the subsequent dot-blot results show that BC-MAP (Y13W and Y79W) and BC-MAP (Y13F and Y79F) retained their antibody binding ability even after mutations at two amino acids. Moreover, we analyzed the binding affinities of the two mutant BC-MAP proteins toward four different isotypes (IgG1, IgG2a, IgG2b, and IgG3) and immune-checkpoint inhibitor CTLA-4 antibody using SPR (Figure 3 and Figure 4). The *K*_D_ values of BC-MAP (Y13W and Y79W) for IgG1, IgG2a, IgG2b, IgG3, and the CTLA-4 antibody were 0, 113, 50.2, 5.49, and 59.1 nM (Figure 5a) and 35, 700, 360, 619.93, and 46 nM for BC-MAP (Y13F and Y79F) (Figure 5b), respectively. These results indicate that both BC-MAP mutants have distinct binding affinities toward various Ig isotypes. Based on these phenomena and the reversible antibody binding affinity of the BC domains, we anticipated that pre-bound CTLA-4 antibodies could be released from the BC domains in response to endogenous antibodies after inserting a BC-MAP-based MN patch with CTLA-4 antibodies into the skin. Because the BC domain exhibits reversible binding depending on the antibody isotype, a sufficiently significant difference in binding affinity between isotypes could result in strong binding to a particular isotype, thereby limiting competitive exchange. Compared to the BC-MAP (Y13F and Y79F), BC-MAP (Y13W and Y79W) exhibited only minor differences in binding affinity (Figure 5c). This minor affinity difference allows for more vigorous competitive interactions between the antibodies, thereby facilitating the efficient delivery of the CTLA-4 antibody. BC-MAP (Y13W and Y79W) was therefore used in subsequent experiments.

### 3.2. Fabrication of the BC-MAP@SF Core–Shell Hydrogel MN Patch Comprising

For transdermal drug delivery applications, MN patches must simultaneously exhibit high biocompatibility and sufficient mechanical strength for reliable skin insertion [22]. Therefore, we constructed the shell layer (responsible for direct antibody presentation) and the core layer (which governs the mechanical rigidity of the MNs) using distinct biomaterials (Figure 1). Specifically, the shell was composed of BC–MAP blended with 20% SF, while the core was formed using 50% SF. Furthermore, a di-tyrosine crosslinking strategy was applied to induce hydrogel formation and ensure cohesive integration between the two layers. This bioinspired reaction forms covalent bonds between tyrosine residues in MAP and SF under visible light, thereby enhancing the mechanical integrity of the MN patch [23].

**Figure 6:**
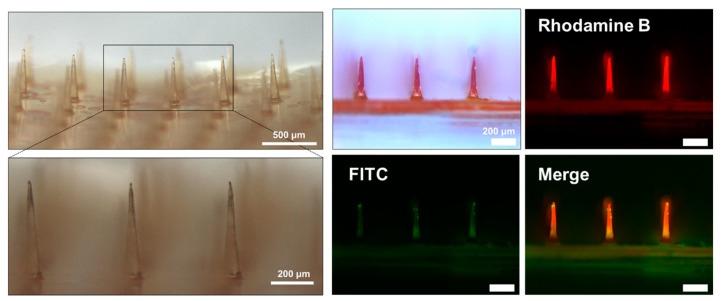
Optical and fluorescence images of core–shell structured BC-MAP@SF MN patch.

The successful fabrication of the BC-MAP@SF core–shell MN patch was confirmed via optical and fluorescence microscopy. Rhodamine B and FITC-dextran incorporated into the shell and core layers, respectively, provided distinct fluorescence signals that verified the well-defined layered architecture of the MNs (Figure 6).

**Figure 7:**
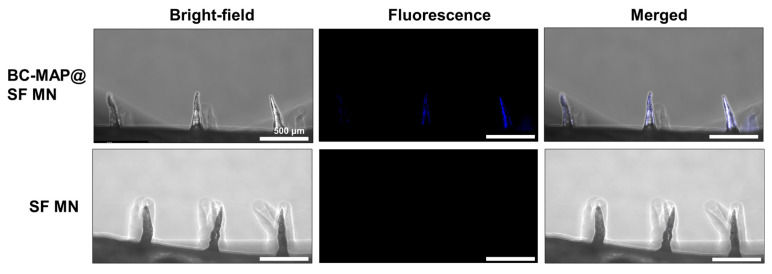
Fluorescence images of Alexa350-antibody-treated BC-MAP@SF MN patch and SF MN patch as negative control.

To evaluate the antibody-binding capability of the BC-MAP@SF MN patch, we incubated it and an SF-only MN patch (as a negative control) with Alexa350-labeled antibodies. As shown in Figure 7, the BC-MAP@SF MN patch exhibited a pronounced blue fluorescence signal localized specifically in the microneedle region. In contrast, no detectable fluorescence was observed in the patch base. In contrast, the SF-only MN patch showed minimal fluorescence. These results indicate that antibodies can selectively interact with the BC domains on the MN surface, thus confirming that the BC-MAP shell retains its antibody-binding functionality following patch fabrication.

### 3.3. Swelling and Mechanical Properties of the BC-MAP@SF Core–Shell Hydrogel MN Patch

**Figure 8:**
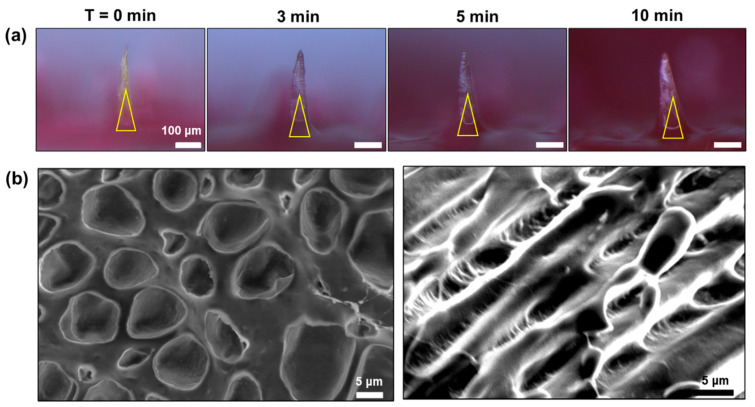
Swelling test of BC-MAP@SF MN patch. (**a**) Morphological changes and (**b**) SEM images of cross-sectioned needle layer. The yellow triangle indicates the core-layer of microneedle.

The swelling property of the BC-MAP@SF MN patch was evaluated to verify whether the circulation of body fluids and antibodies is interrupted after it has been inserted into the skin. Time-dependent morphological changes in the MNs were monitored by using a microscope (Figure 8a). After incubation in PBS, the needle area swelled in a time-dependent manner, with the SEM image of a cross-section of a lyophilized BC-MAP@MN patch in Figure 8b confirming a porous 3D network therein. These results indicate that when the MN patch is inserted into the skin, body fluids can infiltrate the MN’s 3D network, thereby inducing swelling in the needle area, and various biomolecules can migrate between the interior and exterior of the MN through the pores [24,25]. Consequently, we anticipated that a target antibody (e.g., anti-CTLA-4) would be released from the MN through competitive interactions with endogenous antibodies.

**Figure 9:**
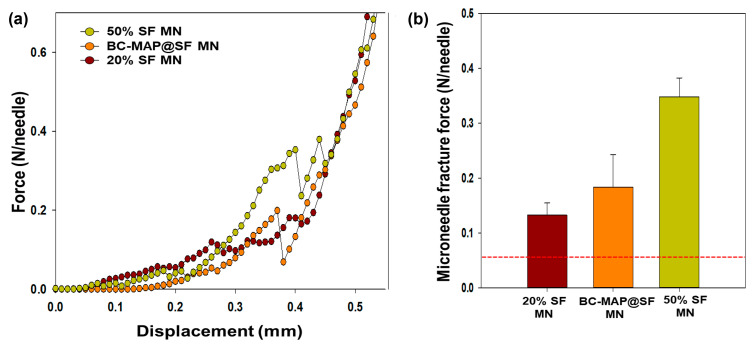
Mechanical property of BC-MAP@SF MN. (**a**) Compression force-displacement curves for 20% SF MN, BC-MAP@SF MN, and 50% SF MN patch (**b**) Ultimate MN fracture forces of 20% SF MN, BC-MAP@SF MN, and 50% SF MN patch obtained by the compression test (*n* = 4). The red dashed line indicates the reported force required for porcine skin penetration (0.05 N/needle).

For efficient transdermal drug delivery, an MN patch must be sufficiently rigid to penetrate the skin [26]. Compression testing determined that the fracture force of the BC-MAP@SF MN patch was 0.21 ± 0.04 N/needle (Figure 9). Moreover, an MN patch composed of 50% SF exhibited a higher fracture force than one composed of 20% SF. The mechanical strength of MNs is influenced by their geometry as well as the ultimate stress of the material [27]. Higher concentrations of silk fibroin result in a denser network, exhibit stronger compressive resistance, and are sufficiently robust to penetrate skin [12]. Moreover, if the material used in the core of the MN patch is rigid and relatively non-swelling, it can provide a stable penetration force during the skin insertion [28]. The BC-MAP@SF core–shell hydrogel MN patch exhibited higher mechanical strength than patches composed solely of 20% SF or BC-MAP MNs owing to the use of 50% SF in the core layer, which instilled relatively high stiffness. The fracture force of the BC-MAP@SF MN patch was over four times greater than that required for porcine skin penetration (0.05 N/needle) [11]. The force required to penetrate human skin has been reported to be 0.08–3.04 N per needle, and porcine skin has approximately 2.5 times the resistance of human skin [15,29]. We assumed that the fabricated MN patch possesses sufficient mechanical strength to penetrate human skin and deliver antibodies.

### 3.4. In Vitro Cytocompatibility of the BC-MAP@SF Core–Shell Hydrogel MN Patch

**Figure 10:**
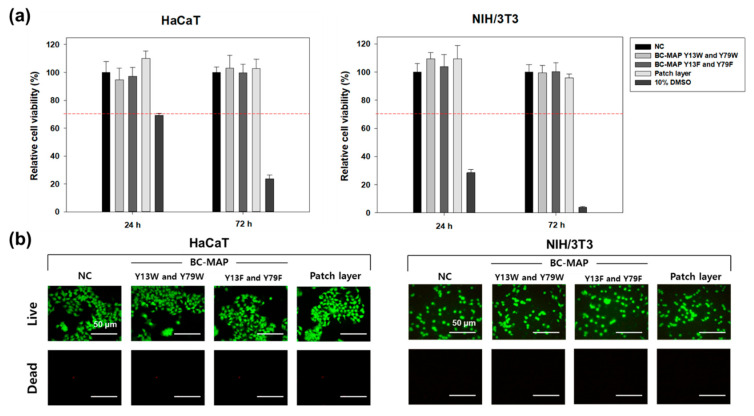
In vitro cytocompatibility of BC-MAP@SF MN patch. (**a**) Relative cell viability of BC-MAP, SF, and 10% DMSO-treated HaCaT and NIH/3T3 cells (*n* = 5). The red dashed line indicates a 30% reduction in cell viability. (**b**) Fluorescence images of HaCaT and NIH/3T3 cells after 3 days of incubation with BC-MAP, SF, and 10% DMSO. The cells were stained using the Live/Dead assay kit.

To assess the cytocompatibility of the BC-MAP@SF core–shell hydrogel MN patch, we conducted a biological evaluation of this medical device via in vitro cytotoxicity testing (ISO 10993-5). Two types of skin cells (HaCaT and NIH3T3) were cultured in media containing extracts from the BC-MAP- and SF-based gels. After 24 and 72 h of culturing, over 90% of the NIH3T3 and HaCaT cells remained viable (Figure 10a). According to the test method, a sample extract is considered biocompatible if cell viability exceeds 70%. Therefore, the BC-MAP@SF hydrogel MN patch is determined to be biocompatible [30,31]. In addition, we verified cell viability using a live/dead staining kit; the fluorescence images in Figure 10b show that most cells emitted a green signal (indicating that they were alive), while very few emitted red signals (indicating dead cells). These findings demonstrate the high biocompatibility of the protein-based MN patch.

## 4. Conclusions

For efficient transdermal antibody delivery, we designed a core–shell hydrogel MN patch that combines the reversible-antibody binding ability of the B and C domains in *S. aureus* protein A with the mechanical strength of SF. The BC-MAP@SF hydrogel MN patch could successfully encapsulate antibodies within the needle region and demonstrated sufficient mechanical strength for human skin insertion. Swelling testing showed a significant expansion of the 3D hydrogel network, which could facilitate the diffusion of endogenous antibodies and the release of pre-loaded antibodies. Although further in vitro and in vivo studies are needed to confirm this mechanism, the overall results suggest that the BC-MAP@SF MN patch has excellent biocompatibility and shows potential as a next-generation platform for transdermal antibody-based immunotherapy.

## Data Availability

The original contributions presented in this study are included in the article. Further inquiries can be directed to the corresponding author.
